# Pourquoi le peau à peau est-il toujours largement méconnu dans nos services de maternité?

**DOI:** 10.11604/pamj.2024.48.177.44729

**Published:** 2024-08-14

**Authors:** Saadi Rania, Saad Benali, Jaouad Kouach, Fatima Ezzahrae Laabidi, Fatima Zahra Kham

**Affiliations:** 1 Service de Gynécologie-Obstétrique, Hôpital Militaire d’Instruction Mohamed V, Université Mohamed V, Rabat, Maroc

**Keywords:** Peau à peau, méthode kangourou, mère, nouveau-né, intérêt, bienfaits, post-partum

## Aux éditeurs de Pan African Medical Journal

De plus en plus de maternités sont adeptes du peau à peau (PAP), une pratique consistant à placer le nouveau-né, dès sa naissance, à même la peau de sa maman, poitrine contre poitrine [1], lui offrant une douce transition entre sa vie intra- et extra-utérine. Il relève de la méthode mère kangourou, qui ciblait initialement les prématurés et les nouveau-nés à faible poids de naissance, dans certains centres hospitaliers confrontés à une pénurie d´incubateurs [2].

Cette méthode aux avantages pourtant largement documentés, reste jusqu´à aujourd´hui peu répandue et assez hétérogène dans les maternités africaines. Cette pratique est pourtant prônée par l'OMS en raison de ses contributions avérées à la thermorégulation du nouveau-né [3], à la régulation de sa glycémie [4], à l'allaitement maternel, ainsi qu´à la prévention des infections [5]. Chez la mère, il réduit le risque d´hémorragie de la délivrance [3] et celui de la dépression du postpartum [6]. S´il ciblait initialement les nouveau-nés de faible poids lors de son introduction en Colombie à la fin des années 70, le contact peau à peau (CPAP) est désormais recommandé pour tous les nouveau-nés indépendamment de l´âge gestationnel, du poids ou du mode d´accouchement.

La vaste majorité des bénéfices du PAP sont principalement imputables à l'influence d'une hormone bien établie en obstétrique: l'ocytocine. Celle-ci est libérée en réponse à la stimulation cutanée et aux mouvements du nouveau-né rampant sur la poitrine de sa mère [7]. En plus de son effet reconnu sur la délivrance placentaire et la rétraction utérine, l'ocytocine entraîne, une élévation de la température maternelle par effet vasodilatateur local. Il en résulte une élévation de la température du nouveau-né, réduisant le risque d'hypothermie et favorisant la conservation d´énergie, le protégeant également d´une éventuelle hypoglycémie [8]. Qui plus est, la production de lait est stimulée par la libération de prolactine induite elle-même par la libération d´ocytocine par les nerfs régionaux. Le PAP trouve ses origines dans la pratique médicale colombienne du 20^e^ siècle. En 1978, deux pédiatres, Edgar Rey Sanabria et Hector Martinez Gomez ont proposé qu´on mette le prématuré nu contre la poitrine de sa mère. Il devait alors être nourri exclusivement au sein avec en supplément du jus de goyave, jusqu'à ce que sa stabilité permette une sortie précoce. Cette technique apparaissait comme une solution palliative de certains problèmes de l´époque comme le manque d'incubateurs, les infections nosocomiales, les taux élevés de mortalité néonatale et d´abandon parental.

Les résultats étaient spectaculaires. En se référant à la première évaluation du Dr Martinez de 1979 à 1994, on a noté une réduction de la mortalité passant de 60% en moyenne à 3,5% sur une étude réalisée sur 467 enfants de même qu´une diminution significative du taux d´infections nosocomiales et d'abandon des enfants [9]. L´efficacité de la technique a conduit à sa validation par l'UNICEF. Toutefois, son adoption dans le monde ne fait pas l'unanimité. Chaque pays s´adapte en fonction de ses réalités.

Dans les pays défavorisés, le plateau technique est déficient et les unités de néonatologie sont en nombre insuffisant pour couvrir le nombre croissant de naissances. Cette situation justifie l´implémentation de la technique du PAP ou de la méthode mère kangourou dans sa globalité, comme solution idoine pour faciliter un tant soit peu l´adaptation à la vie extra-utérine au nouveau-né. D'importantes prouesses sont révélées par le rapport publié en 2012 par l'institution « Save the Children » sur l´impact de la méthode mère kangourou sur le pronostic vital des nouveau-nés, en particulier prématurés, au Mali, Malawi, Ouganda et Rwanda. L'appréciation s´est faite à travers l´implémentation du programme Kangourou dans lesdites régions [10]. Malheureusement, dans la majorité des pays défavorisés, pourtant plus susceptibles d'en bénéficier pleinement, le CPAP demeure sous-utilisé.

Dans les pays développés, la technique du PAP était initialement très peu employée du fait de la disponibilité de moyens sophistiqués pour les soins néonataux. Elle a néanmoins connu un essor ces dernières années, notamment dans le cadre de la démédicalisation de l'accouchement et de la réduction du stress lié à la naissance. Pendant qu´en France, le PAP se proposait aux bébés dont le pronostic vital n´était pas en jeu, il devenait systématique chez les prématurés stables cliniquement dans l´unité de néonatologie du centre universitaire d'Amsterdam [9]. Le PAP intéresse alors aussi bien les bébés à terme que les prématurés [11] ou faibles poids de naissance. Chez ces deux derniers, il est d´ailleurs très préconisé pour une amélioration de leurs paramètres vitaux [12] et pour un bon développement aussi bien moteur qu'intellectuel.

Le CPAP est, tel que susmentionné, recommandé immédiatement après la naissance, les routines de soins néonatales sont par conséquent limitées aux procédures indispensables. Ainsi, si l'état de santé de la dyade mère-enfant le permet, le PAP peut être pratiqué avant le transfert du nouveau-né en néonatologie/nurserie, soit dans la salle d´accouchement, après séchage, au décours d´un accouchement par voie basse ou d´une césarienne [13]. Les bébés séjournant en unité de soins intensifs néonatals peuvent également en bénéficier [14]. Idéalement, le CPAP doit être réalisé en continu durant les deux heures suivant l'accouchement. Pour ce qui est du prématuré, le PAP est maintenu autant que faire se peut, jusqu´à 24 heures sur 24, hormis pour le changement de couche, cette position étant compatible avec l´allaitement maternel.

Si l'impact positif du PAP et sa versatilité ne sont aujourd'hui plus à démontrer, la population africaine ne semble pourtant pas très sensibilisée à cette pratique. Ce qui soulève la question suivante: pourquoi une pratique sans frais, d'exécution simple et aux bénéfices irréfutables était-elle toujours méconnue dans nos services de maternités?
